# Bis(homopiperazine-1,4-diium) cyclo­tetra­phosphate–telluric acid (1/2)

**DOI:** 10.1107/S1600536810038225

**Published:** 2010-10-02

**Authors:** Hanène Hemissi, Mohammed Rzaigui, Salem S. Al-Deyab

**Affiliations:** a Laboratoire de Chimie des Matériaux, Faculté des Sciences de Bizerte, 7021 Zarzouna Bizerte, Tunisia; b Petrochemical Research Chair, College of Science, King Saud University, Riyadh, Saudi Arabia

## Abstract

The title compound, 2C_5_H_14_N_2_^2+^·P_4_O_12_^4−^·2Te(OH)_6_, involves doubly protonated homopiperazinium cations, cyclo­tetra­phosphate anions and telluric acid mol­ecules. The framework possesses very large channels wherein the organic cations reside. A network of O—H⋯O, N—H⋯O and C—H⋯O hydrogen bonds consolidates the crystal packing.

## Related literature

For the properties of materials containing telluric acid, see: Chabchoub *et al.* (2006 ▶); Khemakhem, (1999 ▶). For related structures containing phosphate rings and telluric acid, see: Averbuch-Pouchot & Durif (1987*a* ▶,*b* ▶); Durif *et al.* (1982 ▶). For hydrogen bonding, see: Blessing (1986 ▶); Brown (1976 ▶). For deviations in four-membered phosphate rings having the same 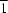 inter­nal symmetry, see: Durif (1995 ▶); A similar conformation for the same organic mol­ecule was observed in (C_5_H_14_N_2_)(H_2_AsO_4_)_2_, see: Wilkinson & Harrison (2006 ▶). For the synthesis, see: Ondik (1964 ▶).
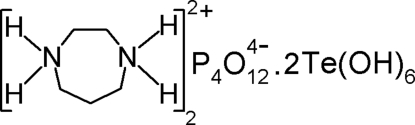


## Experimental

### 

#### Crystal data

C_5_H_14_N_2_^2+^·0.5P_4_O_12_^4−^·Te(OH)_6_
*M* *_r_* = 489.77
Monoclinic, 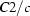

*a* = 20.826 (3) Å
*b* = 8.3600 (13) Å
*c* = 17.030 (8) Å
β = 101.65 (3)°
*V* = 2903.9 (14) Å^3^
*Z* = 8
Ag *K*α radiation
λ = 0.56083 Å
μ = 1.24 mm^−1^
*T* = 293 K
0.2 × 0.18 × 0.16 mm

#### Data collection

Enraf–Nonius CAD-4 diffractometer
8219 measured reflections
6346 independent reflections
4847 reflections with *I* > 2σ(*I*)
*R*_int_ = 0.027
2 standard reflections every 120 min  intensity decay: 5%

#### Refinement

*R*[*F* ^2^ > 2σ(*F* ^2^)] = 0.033
*wR*(*F* ^2^) = 0.084
*S* = 1.01
6346 reflections
223 parameters
12 restraints
H atoms treated by a mixture of independent and constrained refinement
Δρ_max_ = 1.99 e Å^−3^
Δρ_min_ = −1.19 e Å^−3^

### 

Data collection: *CAD-4 EXPRESS* (Enraf–Nonius, 1994 ▶); cell refinement: *CAD-4 EXPRESS*; data reduction: *XCAD4* (Harms & Wocadlo, 1996 ▶); program(s) used to solve structure: *SHELXS97* (Sheldrick, 2008 ▶); program(s) used to refine structure: *SHELXL97* (Sheldrick, 2008 ▶); molecular graphics: *ORTEP-3 for Windows* (Farrugia, 1997 ▶); software used to prepare material for publication: *WinGX* (Farrugia, 1999 ▶).

## Supplementary Material

Crystal structure: contains datablocks I, global. DOI: 10.1107/S1600536810038225/bv2159sup1.cif

Structure factors: contains datablocks I. DOI: 10.1107/S1600536810038225/bv2159Isup2.hkl

Additional supplementary materials:  crystallographic information; 3D view; checkCIF report

## Figures and Tables

**Table 1:** Hydrogen-bond geometry (Å, °)

*D*—H⋯*A*	*D*—H	H⋯*A*	*D*⋯*A*	*D*—H⋯*A*
O1—H1⋯O12^i^	0.86 (2)	1.85 (2)	2.686 (3)	165 (2)
N1—H1*B*⋯O4^ii^	0.90	2.15	2.898 (3)	140
N1—H1*B*⋯O9^iii^	0.90	2.42	3.002 (3)	123
N1—H1*A*⋯O2^iv^	0.90	1.89	2.763 (3)	162
O2—H2⋯O9^v^	0.86 (2)	1.83 (2)	2.688 (3)	172 (3)
N2—H2*A*⋯O12	0.90	1.89	2.769 (3)	167
N2—H2*B*⋯O11^vi^	0.90	1.85	2.739 (3)	172
O3—H3⋯O8^v^	0.84 (3)	2.05 (3)	2.881 (3)	170 (4)
O4—H4⋯O1^vii^	0.85 (3)	1.85 (3)	2.695 (3)	175 (3)
O5—H5⋯O8^viii^	0.87 (3)	1.85 (3)	2.719 (3)	172 (3)
O6—H6⋯O9^ix^	0.83 (3)	1.90 (2)	2.722 (3)	171 (3)
C2—H2*C*⋯O1^x^	0.97	2.60	3.162 (3)	117
C4—H4*B*⋯O5^i^	0.97	2.43	3.256 (4)	142
C5—H5*B*⋯O11^xi^	0.97	2.31	3.265 (4)	168

Symmetry codes: (i) 
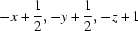
; (ii) 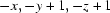; (iii) 
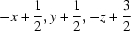
; (iv) 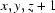; (v) 
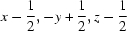
; (vi) 
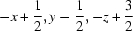
; (vii) 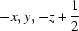; (viii) 
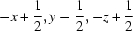
; (ix) 
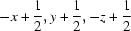
; (x) 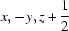; (xi) 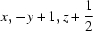.

**Fig. 1.**
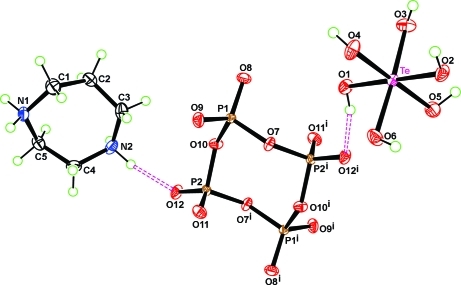
An ORTEP view of (I) with the atom-labelling scheme. Displacement ellipsoids are drawn at the 50% probability level. H atoms are represented as small spheres of arbitrary radii. [Symmetry codes: (i) 1/2 - x, 1/2 - y, 1 - z].

**Fig. 2.**
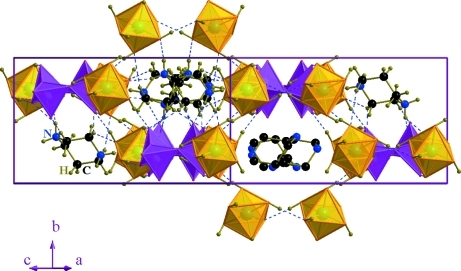
Projection of (I) along [1 0 1] direction. Some hydrogen atoms are omitted for clarity.

**Fig. 3.**
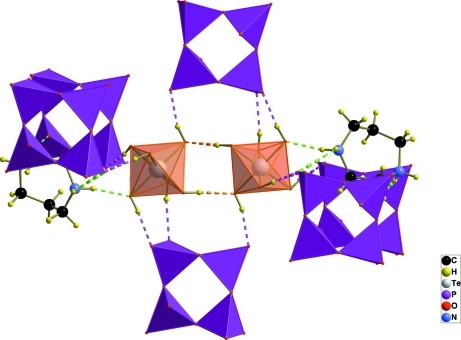
A perspective view of the atomic arrangement of (I).

## supplementary crystallographic information

### Comment

Materials containing telluric acid exhibit many interesting physical properties such as ferroelectricity and protonic conduction (Chabchoub *et al.*,2006; Khemakhem, 1999). On the chemical plane, telluric acid has the property to form adduct compounds with many kinds of phosphates and was extensively investigated during the past thirty years. With cyclotriphosphate, several adduct compounds of telluric acid have been identified whereas with cyclotetraphosphate only three inorganic telluric acid-adduct compounds are reported: (NH_4_)_4_P_4_O_12_.Te(OH)_6_.2H_2_O (Durif *et al.*,1982), K_4_P_4_O_12_.Te(OH)_6_.2H_2_O and Rb_4_P_4_O_12_.Te(OH)_6_.2H_2_O (Averbuch-Pouchot & Durif, 1987*a* b). In the present work, we report the first telluric acid-adduct compound of an organic cyclotetraphosphate (C_5_H_14_N_2_)_2_P_4_O_12_.2Te(OH)_6 _(I). The configuration of different components of (I) is shown in Figure 1. In the crystal structure of this compound, P_4_O_12_^4- ^and Te(OH)_6_ form an anionic three-dimensional framework. The negative charge of this is compensated by the diprotonated amine (C_5_H_14_N_2_)^2+^. Figure 2 shows the total atomic arrangement projected along the [1 0 1] direction. This projection shows that the inorganic entities form very large channels wherein the organic cations are located. The telluric acid molecules Te(OH)_6_ are linked pair-wise by two symmetric strong O—H···O hydrogen bonds, with an O···O distance of 2.695 (3) Å, into dimers where the Te···Te distance is 5.102 (5) Å. The tellurium atom is surrounded by six OH groups in a rather regular octahedral arrangement as indicated by the Te-O distances ranging from 1.905 (2) to 1.916 (2) Å, the *cis* and *trans* O-Te-O angles which are found in the 87.4 (1)-92.94 (9) ° and 175.03 (9)-178.8 (1)° ranges, respectively,  and Te—O—H angles ranging from 107 (3) to 116 (3)°. Each Te(OH)_6_ dimer is linked to six P_4_O_12_ rings and two homopiperazinium (C_5_H_14_N_2_)^2+^ through a three-dimensional hydrogen bonding network O—H···O and N—H···O (Fig. 3). The P_4_O_12_ ring anions are centrosymmetric and are built by only two crystallographically independent PO_4_ tetrahedra with P-O distances ranging from 1.477 (2) to 1.605 (2) Å and O-P-O angles ranging from 103.9 (1) to 120.2 (2)° with a mean value (109.22°) very close to the ideal value (109.28°). In spite of these large ranges in P-O distances and O-P-O angles, which can be explained by different environments of oxygen atoms, the PO_4_ tetrahedron is described by regular oxygen atom arrangement with the phosphorus atom shifted by 0.122 and 0.149 Å from the centre of gravity . The cyclic anion is distorted as evidenced by P—P—P angles (81.60 (1) and 98.40 (1)°) which show a pronounced deviation from the ideal value (90°). Such deviation is commonly observed in tetramembered phosphoric rings having the same -1 internal symmetry (Durif, 1995). Only one homopiperazinium cation (C_5_H_14_N_2_)^2+^ exists in the asymmetric unit and adopts a chair conformation as evidenced by the mean deviation (±0.0251 Å) from the least square plane defined by the four constituent atoms N1, N2, C1 and C3 and the remaining atoms C2, C4 and C5 displaced from the plane by 0.7495 (3), 0.6175 (3) and -0.2661 (3) Å, respectively. A similar conformation for the same organic molecule was observed in (C_5_H_14_N_2_)(H_2_AsO_4_)_2_ (Wilkinson & Harrison, 2006) with important difference that the "seat" chair was defined by one N atom and three C atoms rather than two N atoms and two C atoms as found here. The organic and inorganic components exert between them different interactions (electrostatic, hydrogen bonds and Van der Waals) to form a stable three-dimensional network. The examination of the hydrogen-bond scheme shows the existence of four strong hydrogen bonds with distances O···O ranging from 2.686 (3) to 2.722 (3) Å. The other bonds are weaker, with O(N,*C*)···O distances falling from 2.739 (3) to 3.265 (4) Å (Blessing, 1986; Brown, 1976).

### Experimental

Crystals of the title compound were prepared by adding ethanolic solution (5 ml) of homopiperazine (10 mmol) dropwise to an aqueous solution of cyclotetraphosphoric acid (5 mmol, 20 ml). The obtained solution was added to an aquous solution of telluric acid (10 mmol, 15 ml). Good quality of colourless prisms were obtained after a slow evaporation during few days at ambient temperature. The cyclotetraphosphoric acid H_4_P_4_O_12_, was produced from Na_4_P_4_O_12_.4H_2_O, prepared according to the Ondik process (Ondik, 1964), through an ion-exchange resin in H-state (Amberlite IR 120).

### Refinement

All H atoms of the organic molecule were positioned geometrically and treated as riding on their parent atoms, [N–H = 0.89, C–H = 0.96 Å (CH_3_) with *U*_iso_(H) = 1.5Ueq and C–H =0.96 Å (Ar–H), with *U*_iso_(H) = 1.5Ueq]. The H-atoms of telluric acid,  located in difference Fourier maps, were refined with a distance restraint of O-H = 0.86 (2) Å; their temperature factors were refined.

### Crystal data

**Table:**

C_5_H_14_N_2_^2+^·0.5P_4_O_12_^4^^−^·Te(OH)_6_	*F*(000) = 1936
*M**_r_* = 489.77	*D*_x_ = 2.241 Mg m^−^^3^
Monoclinic, *C*2/*c*	Ag *K*α radiation, λ = 0.56083 Å
Hall symbol: -C 2yc	Cell parameters from 25 reflections
*a* = 20.826 (3) Å	θ = 9.0–10.9°
*b* = 8.3600 (13) Å	µ = 1.24 mm^−^^1^
*c* = 17.030 (8) Å	*T* = 293 K
β = 101.65 (3)°	Prism, colourless
*V* = 2903.9 (14) Å^3^	0.2 × 0.18 × 0.16 mm
*Z* = 8	

### Data collection

**Table:**

Enraf–Nonius CAD-4 diffractometer	*R*_int_ = 0.027
Radiation source: Enraf Nonius FR590	θ_max_ = 27.0°, θ_min_ = 2.1°
graphite	*h* = −33→33
Non–profiled ω scans	*k* = 0→13
8219 measured reflections	*l* = −5→27
6346 independent reflections	2 standard reflections every 120 min
4847 reflections with *I* > 2σ(*I*)	intensity decay: 5%

### Refinement

**Table:**

Refinement on *F*^2^	Primary atom site location: structure-invariant direct methods
Least-squares matrix: full	Secondary atom site location: difference Fourier map
*R*[*F*^2^ > 2σ(*F*^2^)] = 0.033	Hydrogen site location: inferred from neighbouring sites
*wR*(*F*^2^) = 0.084	H atoms treated by a mixture of independent and constrained refinement
*S* = 1.01	*w* = 1/[σ^2^(*F*_o_^2^) + (0.0482*P*)^2^] where *P* = (*F*_o_^2^ + 2*F*_c_^2^)/3
6346 reflections	(Δ/σ)_max_ = 0.001
223 parameters	Δρ_max_ = 1.99 e Å^−^^3^
12 restraints	Δρ_min_ = −1.19 e Å^−^^3^

### Special details

**Table:**

Geometry. All e.s.d.'s (except the e.s.d. in the dihedral angle between two l.s. planes) are estimated using the full covariance matrix. The cell e.s.d.'s are taken into account individually in the estimation of e.s.d.'s in distances, angles and torsion angles; correlations between e.s.d.'s in cell parameters are only used when they are defined by crystal symmetry. An approximate (isotropic) treatment of cell e.s.d.'s is used for estimating e.s.d.'s involving l.s. planes.
Refinement. Refinement of *F*^2^ against ALL reflections. The weighted *R*-factor *wR* and goodness of fit *S* are based on *F*^2^, conventional *R*-factors *R* are based on *F*, with *F* set to zero for negative *F*^2^. The threshold expression of *F*^2^ > σ(*F*^2^) is used only for calculating *R*-factors(gt) *etc*. and is not relevant to the choice of reflections for refinement. *R*-factors based on *F*^2^ are statistically about twice as large as those based on *F*, and *R*- factors based on ALL data will be even larger.

### Fractional atomic coordinates and isotropic or equivalent isotropic displacement parameters (Å^2^)

**Table:**

	*x*	*y*	*z*	*U*_iso_*/*U*_eq_	
C5	0.17222 (12)	0.3358 (3)	0.92873 (16)	0.0227 (5)	
H5A	0.1782	0.2247	0.9450	0.027*	
H5B	0.1909	0.4014	0.9747	0.027*	
C4	0.20920 (13)	0.3672 (3)	0.86247 (18)	0.0253 (5)	
H4A	0.1880	0.4538	0.8291	0.030*	
H4B	0.2535	0.4010	0.8860	0.030*	
H3	−0.0202 (13)	0.058 (5)	0.080 (2)	0.060 (14)*	
H4	−0.0415 (18)	0.298 (4)	0.1851 (19)	0.039 (11)*	
H1	0.1244 (7)	0.217 (4)	0.2504 (13)	0.027 (9)*	
H6	0.0994 (18)	0.505 (3)	0.1086 (14)	0.053 (13)*	
H2	−0.0171 (9)	0.333 (4)	0.0087 (11)	0.024 (8)*	
H5	0.1230 (13)	0.094 (3)	0.068 (2)	0.047 (12)*	
Te1	0.053963 (7)	0.257312 (16)	0.128185 (8)	0.01399 (4)	
P1	0.35495 (3)	0.28147 (6)	0.49522 (3)	0.01199 (9)	
P2	0.26836 (3)	0.30733 (7)	0.61119 (3)	0.01214 (9)	
O10	0.32281 (8)	0.22730 (19)	0.56907 (10)	0.0161 (3)	
O7	0.29316 (8)	0.3056 (2)	0.42301 (10)	0.0167 (3)	
O9	0.39402 (8)	0.1419 (2)	0.47840 (11)	0.0199 (3)	
O1	0.08267 (8)	0.2069 (2)	0.23924 (11)	0.0200 (3)	
O11	0.25530 (8)	0.4740 (2)	0.58395 (11)	0.0197 (3)	
N2	0.21224 (11)	0.2239 (2)	0.81152 (15)	0.0223 (4)	
H2A	0.2422	0.2419	0.7812	0.027*	
H2B	0.2264	0.1404	0.8438	0.027*	
O4	−0.02504 (9)	0.3469 (2)	0.15028 (12)	0.0239 (4)	
O6	0.09666 (11)	0.4585 (2)	0.15090 (12)	0.0273 (4)	
O8	0.38702 (8)	0.4392 (2)	0.51103 (11)	0.0197 (3)	
O12	0.28855 (8)	0.2710 (2)	0.69789 (10)	0.0217 (3)	
O2	0.02429 (9)	0.3119 (3)	0.01751 (11)	0.0229 (4)	
O5	0.13208 (8)	0.1739 (3)	0.10185 (12)	0.0254 (4)	
C3	0.14949 (14)	0.1782 (3)	0.75778 (16)	0.0268 (5)	
H3A	0.1564	0.0813	0.7292	0.032*	
H3B	0.1368	0.2621	0.7183	0.032*	
N1	0.10076 (10)	0.3697 (3)	0.90552 (13)	0.0211 (4)	
H1A	0.0817	0.3340	0.9451	0.025*	
H1B	0.0955	0.4765	0.9031	0.025*	
C2	0.09384 (13)	0.1504 (3)	0.80154 (17)	0.0245 (5)	
H2C	0.0595	0.0911	0.7665	0.029*	
H2D	0.1099	0.0841	0.8481	0.029*	
O3	0.01479 (9)	0.0496 (2)	0.11451 (13)	0.0253 (4)	
C1	0.06439 (13)	0.3009 (4)	0.82876 (18)	0.0289 (5)	
H1C	0.0619	0.3812	0.7871	0.035*	
H1D	0.0199	0.2775	0.8344	0.035*	

### Atomic displacement parameters (Å^2^)

**Table:**

	*U*^11^	*U*^22^	*U*^33^	*U*^12^	*U*^13^	*U*^23^
C5	0.0248 (11)	0.0218 (10)	0.0203 (11)	0.0009 (9)	0.0014 (9)	−0.0054 (9)
C4	0.0217 (10)	0.0176 (10)	0.0379 (15)	−0.0035 (8)	0.0093 (10)	−0.0031 (10)
Te1	0.01393 (6)	0.01490 (6)	0.01358 (6)	0.00039 (5)	0.00382 (4)	0.00058 (5)
P1	0.0102 (2)	0.0138 (2)	0.0119 (2)	−0.00096 (16)	0.00219 (17)	−0.00043 (17)
P2	0.0121 (2)	0.0149 (2)	0.0091 (2)	−0.00041 (17)	0.00137 (17)	−0.00098 (18)
O10	0.0179 (7)	0.0176 (7)	0.0150 (7)	0.0030 (5)	0.0081 (6)	0.0033 (5)
O7	0.0144 (6)	0.0184 (7)	0.0153 (7)	−0.0029 (5)	−0.0016 (5)	0.0042 (6)
O9	0.0178 (7)	0.0238 (8)	0.0187 (8)	0.0042 (6)	0.0051 (6)	−0.0050 (6)
O1	0.0178 (7)	0.0266 (8)	0.0151 (7)	0.0017 (6)	0.0023 (6)	0.0054 (6)
O11	0.0218 (8)	0.0140 (7)	0.0224 (8)	0.0010 (6)	0.0023 (7)	−0.0022 (6)
N2	0.0252 (9)	0.0183 (8)	0.0275 (11)	0.0030 (7)	0.0152 (9)	0.0046 (7)
O4	0.0236 (8)	0.0291 (9)	0.0213 (8)	0.0106 (7)	0.0101 (7)	0.0080 (7)
O6	0.0435 (11)	0.0192 (8)	0.0191 (9)	−0.0103 (8)	0.0060 (8)	−0.0019 (7)
O8	0.0191 (7)	0.0189 (7)	0.0198 (8)	−0.0068 (6)	0.0010 (6)	−0.0013 (6)
O12	0.0173 (7)	0.0394 (10)	0.0084 (6)	−0.0014 (7)	0.0021 (6)	0.0014 (6)
O2	0.0170 (7)	0.0376 (10)	0.0142 (7)	0.0013 (7)	0.0034 (6)	0.0046 (7)
O5	0.0146 (7)	0.0335 (10)	0.0282 (10)	0.0016 (7)	0.0045 (7)	−0.0091 (8)
C3	0.0350 (13)	0.0285 (12)	0.0182 (11)	0.0024 (10)	0.0083 (10)	−0.0008 (10)
N1	0.0248 (9)	0.0209 (9)	0.0197 (10)	0.0009 (7)	0.0094 (8)	−0.0039 (7)
C2	0.0254 (11)	0.0217 (11)	0.0261 (12)	−0.0026 (9)	0.0042 (10)	−0.0058 (9)
O3	0.0218 (8)	0.0196 (8)	0.0326 (11)	−0.0040 (6)	0.0009 (8)	0.0022 (7)
C1	0.0222 (11)	0.0325 (13)	0.0306 (14)	0.0044 (10)	0.0021 (10)	−0.0068 (11)

### Geometric parameters (Å, °)

**Table:**

C5—N1	1.488 (3)	O7—P2^i^	1.6033 (17)
C5—C4	1.512 (4)	O1—H1	0.855 (13)
C5—H5A	0.9700	N2—C3	1.486 (4)
C5—H5B	0.9700	N2—H2A	0.9000
C4—N2	1.488 (3)	N2—H2B	0.9000
C4—H4A	0.9700	O4—H4	0.846 (18)
C4—H4B	0.9700	O6—H6	0.830 (17)
Te1—O5	1.9050 (18)	O2—H2	0.862 (16)
Te1—O6	1.9050 (18)	O5—H5	0.874 (17)
Te1—O1	1.9119 (19)	C3—C2	1.517 (4)
Te1—O3	1.9124 (18)	C3—H3A	0.9700
Te1—O4	1.9130 (18)	C3—H3B	0.9700
Te1—O2	1.916 (2)	N1—C1	1.488 (4)
P1—O8	1.4784 (17)	N1—H1A	0.9000
P1—O9	1.4831 (17)	N1—H1B	0.9000
P1—O7	1.6028 (18)	C2—C1	1.513 (4)
P1—O10	1.6046 (18)	C2—H2C	0.9700
P2—O11	1.4766 (18)	C2—H2D	0.9700
P2—O12	1.4825 (19)	O3—H3	0.840 (19)
P2—O7^i^	1.6033 (17)	C1—H1C	0.9700
P2—O10	1.6052 (17)	C1—H1D	0.9700
			
N1—C5—C4	113.7 (2)	P1—O10—P2	132.59 (11)
N1—C5—H5A	108.8	P1—O7—P2^i^	131.58 (11)
C4—C5—H5A	108.8	Te1—O1—H1	107.4 (15)
N1—C5—H5B	108.8	C3—N2—C4	115.5 (2)
C4—C5—H5B	108.8	C3—N2—H2A	108.4
H5A—C5—H5B	107.7	C4—N2—H2A	108.4
N2—C4—C5	112.5 (2)	C3—N2—H2B	108.4
N2—C4—H4A	109.1	C4—N2—H2B	108.4
C5—C4—H4A	109.1	H2A—N2—H2B	107.5
N2—C4—H4B	109.1	Te1—O4—H4	116 (3)
C5—C4—H4B	109.1	Te1—O6—H6	110.3 (17)
H4A—C4—H4B	107.8	Te1—O2—H2	109.4 (13)
O5—Te1—O6	89.15 (9)	Te1—O5—H5	110.6 (16)
O5—Te1—O1	92.34 (9)	N2—C3—C2	113.6 (2)
O6—Te1—O1	87.36 (8)	N2—C3—H3A	108.8
O5—Te1—O3	90.15 (9)	C2—C3—H3A	108.8
O6—Te1—O3	175.03 (9)	N2—C3—H3B	108.8
O1—Te1—O3	87.75 (9)	C2—C3—H3B	108.8
O5—Te1—O4	177.38 (9)	H3A—C3—H3B	107.7
O6—Te1—O4	89.95 (9)	C1—N1—C5	117.7 (2)
O1—Te1—O4	90.08 (8)	C1—N1—H1A	107.9
O3—Te1—O4	90.96 (8)	C5—N1—H1A	107.9
O5—Te1—O2	88.67 (9)	C1—N1—H1B	107.9
O6—Te1—O2	91.96 (9)	C5—N1—H1B	107.9
O1—Te1—O2	178.78 (8)	H1A—N1—H1B	107.2
O3—Te1—O2	92.94 (9)	C1—C2—C3	114.8 (2)
O4—Te1—O2	88.91 (8)	C1—C2—H2C	108.6
O8—P1—O9	119.45 (10)	C3—C2—H2C	108.6
O8—P1—O7	106.91 (10)	C1—C2—H2D	108.6
O9—P1—O7	109.72 (10)	C3—C2—H2D	108.6
O8—P1—O10	110.71 (10)	H2C—C2—H2D	107.5
O9—P1—O10	105.15 (10)	Te1—O3—H3	107 (3)
O7—P1—O10	103.86 (9)	N1—C1—C2	115.1 (2)
O11—P2—O12	120.22 (11)	N1—C1—H1C	108.5
O11—P2—O7^i^	111.00 (9)	C2—C1—H1C	108.5
O12—P2—O7^i^	106.65 (10)	N1—C1—H1D	108.5
O11—P2—O10	110.78 (10)	C2—C1—H1D	108.5
O12—P2—O10	106.01 (10)	H1C—C1—H1D	107.5
O7^i^—P2—O10	100.20 (9)		

Symmetry codes: (i) −*x*+1/2, −*y*+1/2, −*z*+1.

### Hydrogen-bond geometry (Å, °)

**Table:**

*D*—H···*A*	*D*—H	H···*A*	*D*···*A*	*D*—H···*A*
O1—H1···O12^i^	0.86 (2)	1.85 (2)	2.686 (3)	165 (2)
N1—H1B···O4^ii^	0.90	2.15	2.898 (3)	140
N1—H1B···O9^iii^	0.90	2.42	3.002 (3)	123
N1—H1A···O2^iv^	0.90	1.89	2.763 (3)	162
O2—H2···O9^v^	0.86 (2)	1.83 (2)	2.688 (3)	172 (3)
N2—H2A···O12	0.90	1.89	2.769 (3)	167
N2—H2B···O11^vi^	0.90	1.85	2.739 (3)	172
O3—H3···O8^v^	0.84 (3)	2.05 (3)	2.881 (3)	170 (4)
O4—H4···O1^vii^	0.85 (3)	1.85 (3)	2.695 (3)	175 (3)
O5—H5···O8^viii^	0.87 (3)	1.85 (3)	2.719 (3)	172 (3)
O6—H6···O9^ix^	0.83 (3)	1.90 (2)	2.722 (3)	171 (3)
C2—H2C···O1^x^	0.97	2.60	3.162 (3)	117
C4—H4B···O5^i^	0.97	2.43	3.256 (4)	142
C5—H5B···O11^xi^	0.97	2.31	3.265 (4)	168

Symmetry codes: (i) −*x*+1/2, −*y*+1/2, −*z*+1; (ii) −*x*, −*y*+1, −*z*+1; (iii) −*x*+1/2, *y*+1/2, −*z*+3/2; (iv) *x*, *y*, *z*+1; (v) *x*−1/2, −*y*+1/2, *z*−1/2; (vi) −*x*+1/2, *y*−1/2, −*z*+3/2; (vii) −*x*, *y*, −*z*+1/2; (viii) −*x*+1/2, *y*−1/2, −*z*+1/2; (ix) −*x*+1/2, *y*+1/2, −*z*+1/2; (x) *x*, −*y*, *z*+1/2; (xi) *x*, −*y*+1, *z*+1/2.
